# The grand challenge of discovering new cardiovascular drugs

**DOI:** 10.3389/fddsv.2022.1027401

**Published:** 2022-09-23

**Authors:** Charles C. Hong

**Affiliations:** Departments of Medicine, Physiology, and Biochemistry and Molecular Biology, Center for Biomolecular Therapeutics, University of Maryland School of Medicine, Baltimore, MD, United States

**Keywords:** drug discovery, heart failure, human genetics, inherited cardiomyopathy, induced pluripotent stem cells, academia-biotech-pharma ecosystem, real world data, biobanking and biorepositories

## Background

Heart disease is the #1 killer worldwide, greater than all cancers combined. This is despite the fact that, in the developed world, there has been a substantial decline in cardiovascular mortality since the mid-20th century (Centers for Disease Control and Prevention (CDC), 1999), driven largely by a reduction in ischemic heart disease (Mensah et al., 2017; Nowbar et al., 2019). This decline is multifactorial, involving a reduction in tobacco use, changes in diet, treatment of hypertension, advances in rapid coronary revascularization, and the advent of β-hydroxy β-methylglutaryl-CoA (HMG-CoA) reductase inhibitors, and P2Y12 ADP receptor antagonists (Arnett et al., 2019). However, with the adoption of the Western diet and lifestyle in the developing world, and the rise in prevalence of cardiometabolic diseases and obesity, there has been an increase in the global burden of cardiovascular diseases (CVD) (Roth et al., 2020) and a stalling of improvements in the United States (Sinatra and Huston, 2020).

Our collective success in treating cardiovascular diseases has been mixed. While the relative burden of ischemic heart disease (IHD) has decreased in the developed world, the burden of heart failure (HF) has increased in the past several decades (Virani et al., 2021). HF is now a global pandemic, afflicting 26 million people worldwide and 6 million in the United States alone (Savarese and Lund, 2017; Jackson et al., 2018). Despite advances in medical and surgical therapies, HF is associated with high mortality, having a lower 5-years survival rate than most cancers (Virani et al., 2021). In fact, even as the mortality rate from IHD dropped 14.9% from 2011 to 2017, the HF mortality rate increased 20.7% during the same period (Sidney et al., 2019). Additionally, HF is associated with significant morbidity, reduced quality of life, and loss of earning potential due to diminished functional capacity. Moreover, advanced HF is frequently associated with anemia, sarcopenia, renal failure, depression, and cognitive decline (Lang and Mancini, 2007). Medical management is difficult and often complicated by volume overload and/or kidney injury. Consequently, HF is a leading diagnosis for hospitalizations and rehospitalization in the United States (McDermott and Roemer, 2021). Even as the advent of goal-directed medical therapy (GDMT) has substantially modified the rate of progression of HF, and internal cardiac defibrillators have reduced the risk of sudden cardiac death (van der Meer et al., 2019), HF remains a progressive disease, often necessitating advanced therapies like intravenous inotropic agents, mechanical support for cardiogenic shock, and ultimately heart transplantation. Finally, mechanical support and heart transplantation can only meet a tiny fraction of the global demand, and for those with end-stage HF who do not get mechanical support or heart transplantation, the 1-year mortality is extremely high (Rose et al., 2001). Thus, HF is among the largest unmet medical need of the 21st century, and developing new drugs for cardiovascular diseases (CVDs), particularly HF, is a Grand Challenge for our generation!

For drug companies, the cost of developing new molecular entities (NMEs) into approved therapies is extremely high, with costs often ranging over $3 billion per launched NME (Schuhmacher et al., 2016). A major reason for this is that only 5–10% of candidate NMEs are eventually approved (Nelson et al., 2015). For those in the cardiovascular drug development field, which often relies on large, expensive clinical trials to demonstrate efficacy, the stakes could not be higher. Given the dual societal needs for both effective new therapies and lower drug costs, new, more efficient paradigms for the discovery and development of cardiovascular and hematologic drugs will help meet this challenge.

**FIGURE 1:**
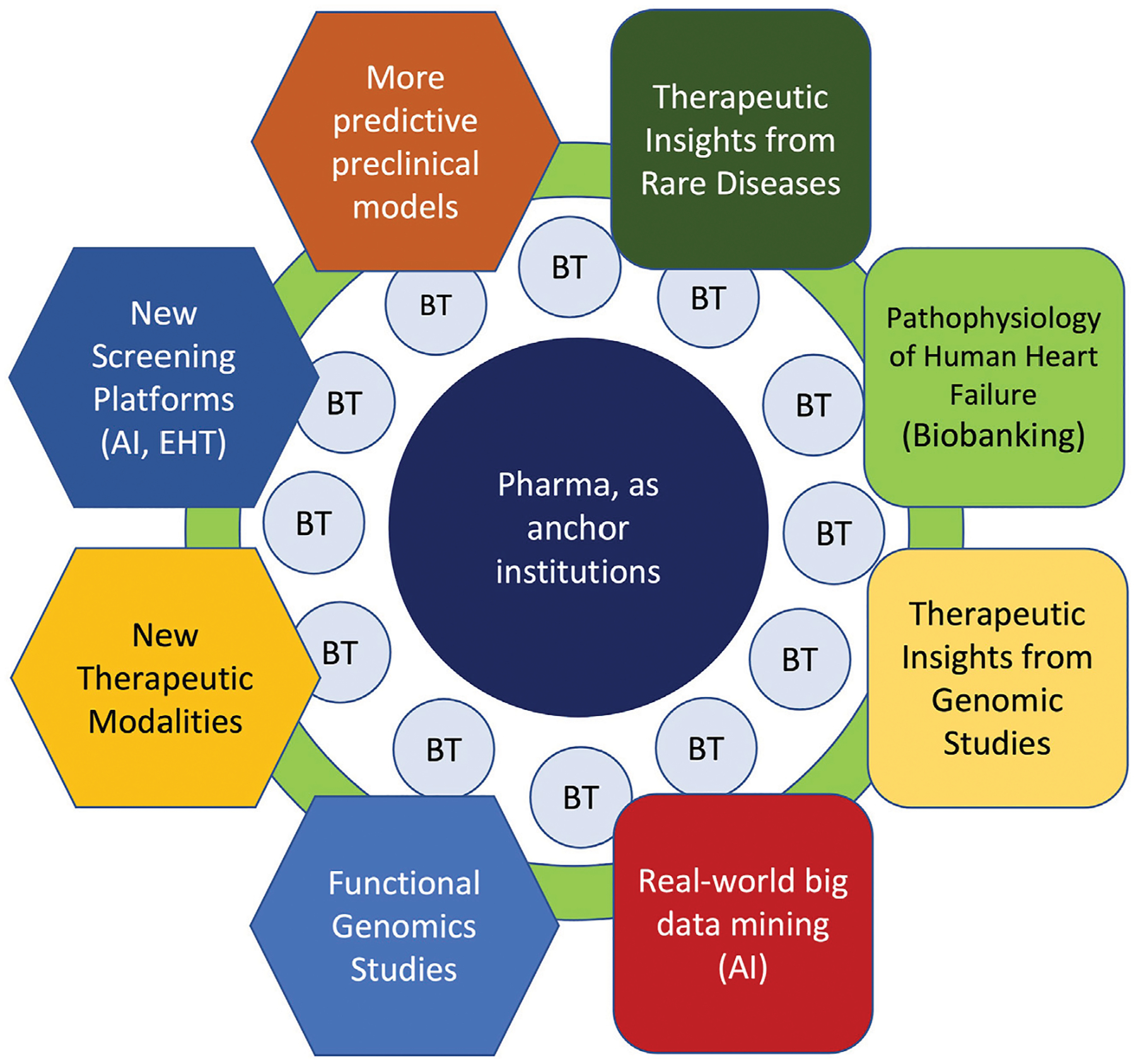
Key Areas for Cardiovascular Drug Discovery Within the Context of Academia-Biotech-Pharma Ecosystem. Successful cardiovascular drug discovery effort will rely on continual progress in academia in basic (hexagons) and translational (rounded squares) research areas to gain deeper mechanistic insights to human heart disease. Moreover, within the academia-biotech-pharma ecosystem, these research areas must cross-fertilize across disciplines to accelerate drug discovery. While much of the fundamental discoveries originate in academia, biotech (BT) milieu facilitates the translation of ideas into products through de-risking in the early stages. By providing seed funding, industry know-how and resources, large pharmaceutical companies can serve as anchor institutions that catalyze innovative ideas into new therapies. Finally, academic institutions play an essential role of training future drug makers.

Those of us in the fields of cardiovascular research and medicine face the paradox of a diminishing drug pipeline in an explosive era of biological discovery. Notwithstanding the recent success of sodium-glucose transport protein 2 (SGLT2) inhibitors for HF, recent analyses indicate a steady decline in the number of new cardiovascular drugs under development between 1990 and 2012 (Hwang et al., 1990). Moreover, many drugs in development during this period, including antihypertensive agents, neurohormonal blockers, lipidmodifying agents and anticoagulants, targeted previously proven mechanisms (Figtree et al., 2021). This situation stands in contrast to the ever-expanding compendium of potential molecular targets and dramatic improvements in small molecule design using artificial intelligence (AI) (Jayatunga et al., 2022). As we launch *Frontiers in Drug Discovery, Cardiovascular Hematologic Drugs*, I want to highlight several key areas that this journal can focus on to tip the scale in favor of success in this domain (Figure 1).

## Better fundamental understanding of human HF

A recent HF compendium highlighted several translational successes, including the exciting prospects for SGLT2 inhibitors for heart failure with reduced ejection fraction (HFrEF) (McMurray et al., 2019) or heart failure with preserved ejection fraction (HFpEF) (Nassif et al., 2021), and tafamidis for transthyretin (TTR) cardiac amyloidosis (Maurer et al., 2018); yet strikingly, only one of the therapeutic approaches covered in several HF compendia was translated into a new therapy for patients with HF (Mann, 2021). The relative paucity of new drug classes for HF is rooted in our incomplete understanding of HF pathophysiology. This in turn stems from the relative lack of access to human cardiovascular tissue samples beyond terminal stages, when fibrosis and scarring predominate, obscuring important processes at play during earlier stages of disease progression. As a result, systems-level evaluation of cell pathways and networks involved in the *pathogenesis* of human HF, which could reveal new therapeutic strategies, is still in infancy. Consequently, although fibrosis is integral to HF progression and cardiac remodeling, it is still unclear whether therapies purposely developed to directly target fibrosis will have beneficial effects on HF (Sweeney et al., 2020). Thus, biobanking efforts to identify biomarkers and molecular signatures of disease progression before progression to end-stage heart disease will aid the development of rational therapeutic strategies for HF (Hahn et al., 2021).

In contrast to the cancer field, where traditional anatomic descriptors have given way to precise molecular descriptors that yield insights as to the drivers of oncogenesis, CVDs tend to be lumped into broad descriptive categories (e.g., HFpEF and HFrEF), with little regard to the underlying pathophysiology. For instance, the significant discrepancy between survival statistics for HFpEF in cross-sectional studies versus clinical trials strongly suggests that the very definition of HFpEF is uncertain and that the term *HFpEF* remains an amalgam of symptomatology (Oktay et al., 2013). Recognition of disease heterogeneity warrants a more mechanism-based subclassification of HFpEF (Shah et al., 2020). Similarly, HFrEF could benefit from reclassification that reflects the diversity of its pathophysiology. For example, the natural history (i.e., clinical outcomes) of HF due to underlying genetic perturbations, such as titin frameshift/truncation mutations, may be intrinsically different from HF without a genetic etiology. The traditional tendency for those in the cardiovascular realm to be “clumpers” served us well during the era of the “low-hanging fruit” of the neurohormonal blockade as a broad disease-modifier for patients with HFrEF, regardless of the specific etiology, but this is akin to oncology before the advent of targeted therapies. Although neurohormonal blockade, including beta-adrenergic receptor, renin-angiotensinaldosterone system and neurolysin inhibitors, has transformed the medical care of HF patients, the number needed to treat (NNT) with each of these agents to prevent one cardiovascular death over 5 years is typically >10 (Srivastava et al., 2018). The big question for the future in our field is how much more can we improve on this?

Given the lack of a full understanding of HF etiology, it is perhaps not too surprising that major treatment advances, such as the neurohormonal blockade and SGLT2 inhibitors, have their roots in serendipity. However, such non-mechanistic approaches necessitate large, expensive clinical trials to demonstrate benefit compared to existing therapies. Few pharmaceutical companies are willing to take such huge risks without compelling preliminary data. Moreover, while SGLT2 inhibitors are indeed groundbreaking, I wonder what the return on investment would have been if HF benefits were recognized earlier. Thus, the HF drug discovery effort could benefit from a systematic process to vet drug candidates for repositioning to HF indications earlier in the drug development process (Pushpakom et al., 2019; Abdelsayed et al., 2022).

## Therapeutic insights from rare diseases

Serendipity alone is not a sustainable strategy for drug development. At a minimum, greater recognition of the heterogeneous etiology of HF and a more mechanism-based method of classification is necessary to advance our field. For example, the advent of tafamidis for TTR amyloid cardiomyopathy represents a triumph of mechanism-based rational drug design. TTR amyloid cardiomyopathy is characterized by progressive deposition of TTR amyloid fibrils. In older individuals, wild-type TTR protein is prone to aggregation, causing cardiac disease, whereas a variety of destabilizing TTR variants can lead to cardiomyopathy or polyneuropathy. In a triumph of structure-based chemical biology, Kelly and others developed tafamidis, which binds to TTR, preventing pathogenic TTR aggregation and amyloidogenesis (Bulawa et al., 2012). While tafamidis is marketed as an “orphan” drug, with a very high price tag, it may ultimately prove to be efficacious for a broader population since the V142I variant, carried by 3.5% of African Americans, is associated with increased rates of incident heart failure and a lower overall survival (Chandrashekar et al., 2021).

Rare inherited hypertrophic cardiomyopathies (HCMs) have revealed promising new therapeutic approaches. HCM is an inherited heart disease characterized by abnormal thickening and fibrosis of the heart, but the fundamental pathophysiology involves excessive cardiac contractility and impaired relaxation. In a biomechanical screen for compounds that reduce sarcomere contractile function, Green and others discovered mavacamten, a negative allosteric modulator of beta cardiac myosin (Green et al., 2016) that was recently approved as a therapy for symptomatic HCM. This study in a mouse HCM model also showed that early, chronic administration of mavacamten suppresses the development of ventricular hypertrophy, cardiomyocyte disarray, and myocardial fibrosis. Although the precise mechanism whereby hyperdynamic contraction leads to HCM pathology is unclear, this study suggests that mavacamten may also be beneficial for the prevention of HCM disease manifestations. A better mechanistic understanding of this pathogenic link may lead to new therapeutic opportunities for expanded indications such as HFpEF. Likewise, a thorough molecular and mechanistic understanding of the pathogenesis of rare inherited dilated cardiomyopathies promises to reveal new therapeutic insights. For example, future therapies for heart failure due to *RBM20*, *LMNA*, and *BAG3* mutations may involve specific strategies that modulate RNA metabolism (Guo et al., 2012; Green et al., 2016; Schneider et al., 2020; Fenix et al., 2021), mechanosensing (Chai et al., 2021) and proteostasis (Meister-Broekema et al., 2018; Ding et al., 2019; Martin et al., 2021), respectively.

## Insights from human genetics and functional genomics

As specific TTR variants that destabilize TTR structures were known to be associated with either amyloid cardiomyopathy or familial amyloid neuropathy, the tafamidis story also highlights the power of human genetics to elucidate viable therapeutic strategies. Given the extraordinary costs and risks of developing NMEs, the pharmaceutical and biotech industries are investing in genomics to improve target selection and decrease the risk of failure from lack of efficacy (Cook et al., 2014) or adverse effects (Nguyen et al., 2019a). Indeed, an analysis of historical pipeline data concluded that pipeline drug targets with human genetic evidence of disease association are twice as likely to lead to approved drugs as those without such evidence (Nelson et al., 2015), and, when the causal linkages are clear (i.e., Mendelian traits linked to coding variants), the use of human genetic evidence increases approval even more (King et al., 2019).

Unbiased genome-wide association studies (GWAS) for coronary artery disease (CAD), for example, have provided a rich collection of dozens of loci that revealed novel therapeutic insights into atherosclerosis (Nurnberg et al., 2016). Since most genetic variants found in GWAS do not affect protein sequences, functional genomic studies that strengthen or refute gene-phenotype associations as well as directionality of effect are vital to prioritize therapeutic targets (Musunuru et al., 2018). Thanks to recent technical advances, there have been numerous functional genomic studies that strengthen biological plausibility originating from genetic studes (Nurnberg et al., 2016). Broadly speaking, technical advances include methods to analyze the impact of a putative causal variant on gene expression, including assessment of expression quantitative trait loci (eQTL), allele-specific expression (ASE), and chromatin status. Additionally, genomic editing, gene replacement and gene knockdown technologies, tissue modeling using human induced pluripotent stem cells (iPSCs) and genetic animal models can provide critical mechanistic and functional insights of putative causal variants at the cellular level and in preclinical models. Finally emerging “high-throughput biology” technologies, such as massively parallel reporter assay (MPRA) to identify functional regulatory variants and transcriptional networks across multiple cell types, can be utilized to identify novel gene-phenotype associations or further strengthen known associations (Cooper et al., 2022).

Cardiovascular drug discovery has benefited greatly from human genetics. For instance, the discovery of PCSK9 (proprotein convertase subtilisin/kexin type 9) loss-of-function mutations, which confer lifelong protection against atherosclerosis and coronary heart disease (CHD) without discernible deficits in homozygous carriers (Cohen et al., 2005), provided a compelling “failsafe rationale” for the development of antibodies and small interfering RNAs (siRNAs) against PCSK9, or even gene editing for the treatment of hypercholesterolemia and prevention of atherosclerosis (Nurnberg et al., 2016; Musunuru et al., 2018; King et al., 2019). Human genetics can also provide early warning signals against drug development efforts that will ultimately prove fruitless. Perhaps, human genetics could have presaged the failures of the cholesteryl ester transfer protein (CETP) inhibitors (Voight et al., 2012). application of human genetics to identify therapeutic targets for HF is much more challenging since HF is a heterogeneous condition with multiple distinct pathologies, and in contrast to lipidomics, there is no unifying biomarker that is causally associated with HF. As discussed below, even larger clinical-genetic datasets and the creative use of phenome-wide association studies (PheWAS) may yield novel therapeutic insights (Denny et al., 2013; Pulley et al., 2017; Challa et al., 2019).

## Search for residual risk and “missing biology”

Figtree and others eloquently outline the need to elucidate the mechanism behind residual risk and “missing biology” (Figtree et al., 2021). These authors highlight five patient groups with unmet needs for therapeutic insights: resistant hypertension; recurrent acute vascular events despite optimal care; atherosclerotic CVD in the absence of known modifiable risk factors; rapid progress of HF despite optimal GDMT; and HFpEF. The concept of residual risk is highlighted by the fact that up to 23% of patients with ST-segment elevation myocardial infarction do not have traditional modifiable risk factors (hypercholesterolemia, diabetes mellitus, hypertension, and smoking) (Vernon et al., 2019), and preventable factors account for only one-half of deaths from CVD (Patel et al., 2015).

Recently, clonal hematopoiesis of indeterminate potential (CHIP), which refers to the expansion of somatic blood-cell clones attributed to acquired mutations in genes such as DNMT3A and TET2, was shown to be associated with prevalent myocardial infarction, nearly doubling of the risk of CHD (Jaiswal et al., 2017), and higher incident heart failure risk (Yu et al., 2021). Interestingly, in individuals with an increased risk of CVD due to large CHIP clones, the presence of a hypofunctional mutation in the interleukin-6 receptor (IL6R; p. Asp358Ala) abrogated CVD events and the risk of myocardial infarction (Bick et al., 2020). These results suggest that anti-inflammatory therapies targeting interleukin-6 (IL6) signaling may attenuate CVD event risk specifically in those with elevated risk due to CHIP. As CHIP is associated with aging, IL6 signaling may represent a novel, personalized means to modify a risk traditionally not thought to be modifiable (i.e., aging). It remains to be seen whether similar therapeutic relationships can be found for HF associated with CHIP.

In terms of human genetics, much of the “missing biology” can be attributed to the lack of racial and ancestry diversity in most GWAS and Mendelian genetic studies and clinical studies in general. For example, less than 2.5% of subjects in 3,700 GWAS studies were of African American, Afro-Caribbean and African ancestry (Mills and Rahal, 2019). The lack of diversity in research, particularly among those of African descent, is ironic since cardiac amyloid and *BAG3*-mediated cardiomyopathy both involve causal variants that are much more prevalent in African American subjects (Shah et al., 2016; Myers et al., 2018). In the CARDIA study, incident heart failure before 50 years of age was 20-times higher among blacks than among whites, representing a huge untapped opportunity to discover the “missing biology” of HF (Bibbins-Domingo et al., 2009). Lastly, a recent large-scale GWAS of coronary artery disease (CAD) in genetically diverse populations of the Million Veteran Program identified 95 novel loci associated with CAD (Tcheandjieu et al., 2022), a stunning tour de force demonstration of the value of studying diverse populations to increase our understanding of cardiovascular disease pathophysiology.

## More predictive preclinical models

The drying up of the cardiovascular drug pipeline in the past several decades is particularly ironic because it came at the same time as the explosion of preclinical studies of CVDs. Thanks to gene knockout technology in mice, the predominant preclinical model, there are now thousands of reports on new potential therapeutic targets relevant to myocardial infarction, ischemia-reperfusion injury, fibrosis, metabolic reprogramming, and HF. However, to date, no approved cardiovascular drug has been developed based exclusively on genetic mouse models. The reasons for this “failure to translate” include fundamental species differences in cell biology and physiology between humans and mice, overreliance on reductionist inbred genetic models, and the underappreciation of sex as a variable. The issue is particularly problematic for cardiac regenerative therapies because human hearts do not regenerate appreciably. Of course, the solution to this translational challenge is not to abandon research on preclinical models. One way to enhance their translational potential is to complement mouse studies with studies on patient-derived human induced pluripotent stem cell (iPSC) models and other animal models such as zebrafish and large animals. Manipulation of evolutionarily conserved biology is more likely to translate to humans.

For therapeutic development, the advancement of predictive preclinical models of human CVDs is of the utmost importance. For HFpEF, Hill and others developed a mouse model involving concomitant metabolic and hypertensive stress elicited by the combination of a high-fat diet and inhibition of constitutive nitric oxide synthase using N (gamma)-nitro-L-arginine methyl ester (L-NAME), which recapitulates the numerous systemic and cardiovascular features of HFpEF in humans (Schiattarella et al., 2019). Importantly, the myocardium of this rodent model and humans with HFpEF shared impaired expression of an unfolded protein response effector, the spliced form of X-box-binding protein 1 (XBP1s). In mice, the inducible nitric oxide synthase (iNOS)-driven inhibition of the IRE1α-XBP1 pathway reproduces HEpEF, and genetic suppression or pharmacological inhibition of iNOS with l-N6-(1-iminoethyl) lysine ameliorated the HFpEF phenotype. While it is widely accepted that HFpEF in man is a heterogenous disease affecting many organ systems (Shah et al., 2020; Hahn et al., 2021), the discovery of HFpEF pathophysiology shared by mice and humans opens up a vital translational path for the first targeted therapy for HFpEF in humans.

## Increased reproducibility in preclinical research: importance of rigorous negative studies

A major issue affecting both preclinical and clinical research is positive reporting bias. Consequently, in drug discovery, although hardly anything works in humans, new therapeutic ideas seemingly endlessly arise from preclinical models. Because only positive studies are rewarded, there is an unhealthy incentive to focus exclusively on exciting new breakthroughs rather than on rigorously validating or refuting earlier findings. To move drug discovery forward as a community, publishing of rigorous “negative studies” by academia and biopharma should be encouraged. As a case in point, Gupta and others recently published a rigorous “negative” functional genomic study of corin, a protease expressed in cardiomyocytes that plays a key role in the activation of natriuretic peptides (Wang et al., 2021). Despite compelling pathophysiology and a prior observation suggesting an association of corin with coronary artery disease, they found no relationship between rare, functionally damaging corin variants or circulating corin concentrations and the risk of coronary artery disease. Such reporting of rigorous studies ruling out the viability of a therapeutic hypothesis is valuable for increasing the collective efficiency of cardiovascular drug discovery.

## New screening platforms

Another challenge to the development of new cardiovascular medicines is the difficulty of screening for novel therapeutic agents due to a lack of suitable models. As discussed, there are important physiological differences between human and murine hearts. For instance, the human heart rate is ~80 beats per minute whereas the mouse heart rate is ~600 beats per minute. The advent of human iPSCs and the ability to make limitless numbers of cardiomyocytes that recapitulate human cardiac physiology could revolutionize heart disease modeling and drug discovery. Although no *in vitro* platform is perfect, there have been tremendous advances, including methods to promote cardiomyocyte maturation (Feaster et al., 2015; Parikh et al., 2017; Feyen et al., 2020; Funakoshi et al., 2021; Knight et al., 2021) and new engineered tissue constructs that could tease out subtle yet important differences between the myotropes Danicamtiv and Omecamtiv mecarbil (Nguyen et al., 2019b; Shen et al., 2021). Leveraging advances in high-content imaging, phenotype-based screens of human iPSC-derived cardiovascular tissues are poised to make a major impact on CV therapeutic discovery (Mills et al., 2019; Briganti et al., 2020; Hnatiuk et al., 2021; Vincent et al., 2022).

## New therapeutic modalities

Although small molecules have dominated the therapeutic landscape for hundreds of years, the advent of antibody therapeutic agents with precise target engagement has dramatically accelerated drug development. A key disadvantage of therapeutic antibodies is that they only target extracellular proteins. In contrast, siRNA and antisense mRNA can selectively modulate their targets, regardless of localization. In recent years, gene therapies have regained momentum, especially for rare genetic diseases (ClinicalTrial.gov # NCT03882437). The spectacular success of modified mRNA and lipid nanoparticles (LNP) for COVID19 vaccines has opened up a promising new option for gene delivery (Collén et al., 2022) and targeted ablation of profibrotic activated fibroblasts (Rurik et al., 2022). Recently, Verve Therapeutics has taken the bold step of applying the latest genomic editing technology to permanently delete the *PCSK9* gene *in vivo* (Musunuru et al., 2021; Musunuru, 2022). Together with advances in organ-targeted LNPs (Cheng et al., 2020), these new modalities have the potential to revolutionize the therapeutic development of human genetics-validated therapeutic targets and facilitate the development of multiple “niche” therapies for specific disease segments.

## Real-world big data for therapeutic hypothesis validation, drug repositioning and repurposing

A potential powerful “search engine” to discover new cardiovascular drugs is the “real-world” data contained within the electronic health records (EHRs) of thousands of patients. For example, the DiscovEHR, a collaboration between the Regeneron Genetics Center and the Geisinger Health System, which combines information from high-throughput DNA sequencing with longitudinal EHRs, has led to the discovery of genetic variations important for human disease and the identification of therapeutic targets (Dewey et al., 2016). For example, this collaboration revealed that genetic inactivation of ANGPTL3 decreased levels of all three major lipid fractions and the risk of ASCVD. Furthermore, pharmacological antagonism of ANGPTL3 phenocopied its genetic inactivation, thus providing powerful evidence that ANGPTL3 is a viable therapeutic target (Dewey et al., 2017).

Vanderbilt University Medical Center (VUMC) has used BioVU, the Medical Center’s massive DNA databank, to look for genetic variations associated with biological changes that resemble drug effects (Pulley et al., 2017). One approach VUMC has pioneered is phenome-wide association studies (PheWAS), in which EHRs are scanned for associations between genetic variations of a single gene and all diseases described in the EHR (Denny et al., 2010; Denny et al., 2013). If genetic variations of a particular gene are associated with a lower incident risk of a disease, one may infer how a lifelong modulation of that gene’s function may be associated with the disease manifestation. By that logic, one may hypothesize that drugs which target that gene product may be useful for that particular condition. Similarly, PheWAS is also useful for identifying potential deleterious effects of engaging these targets (Challa et al., 2019). For a heterogeneous disease like HF, the “gene first” PheWAS approach has the potential to find biologically relevant genotype-phenotype associations that were missed by traditional GWAS. Additionally, drug-wide association studies (DrugWAS) were recently developed to search EHRs for drugs associated with lower incidence or severity of COVID-19 in order to repurpose existing drugs that may confer protection against it (Bejan et al., 2021). One can easily envision how such approaches may be used to identify existing drugs that could be repurposed to reduce cardiovascular morbidities.

When it comes to EHR data, bigger is better. The threat of the COVID-19 pandemic has pushed academic medical centers to form the National COVID Cohort Collaborative (N3C), an EHR repository to help identify features and drugs that may mitigate or exacerbate COVID-19 (Haendel et al., 2021). In just its first year, N3C collected and organized 9.7 billion rows of data from 8.5 million patients at more than 60 medical centers. Among its numerous findings, the N3C found that early aspirin use was associated with lower risk of inpatient death from Covid-19 (Chow et al., 2022). Additionally, the N3C is utilizing AI/machine learning methodologies to analyze the massive real-world data to help identify those with long COVID (Pfaff et al., 2022a). The success of N3C demonstrates that similar large-scale efforts across multiple medical systems and EHR platforms, coupled with “omics” data and AI/ML-based analytics, can be implemented to identify factors, biomarkers and drug targets that impact cardiovascular health (Pfaff et al., 2022b; Salah et al., 2022).

## A synthesis: Proposed path for HF drug discovery

**FIGURE 2:**
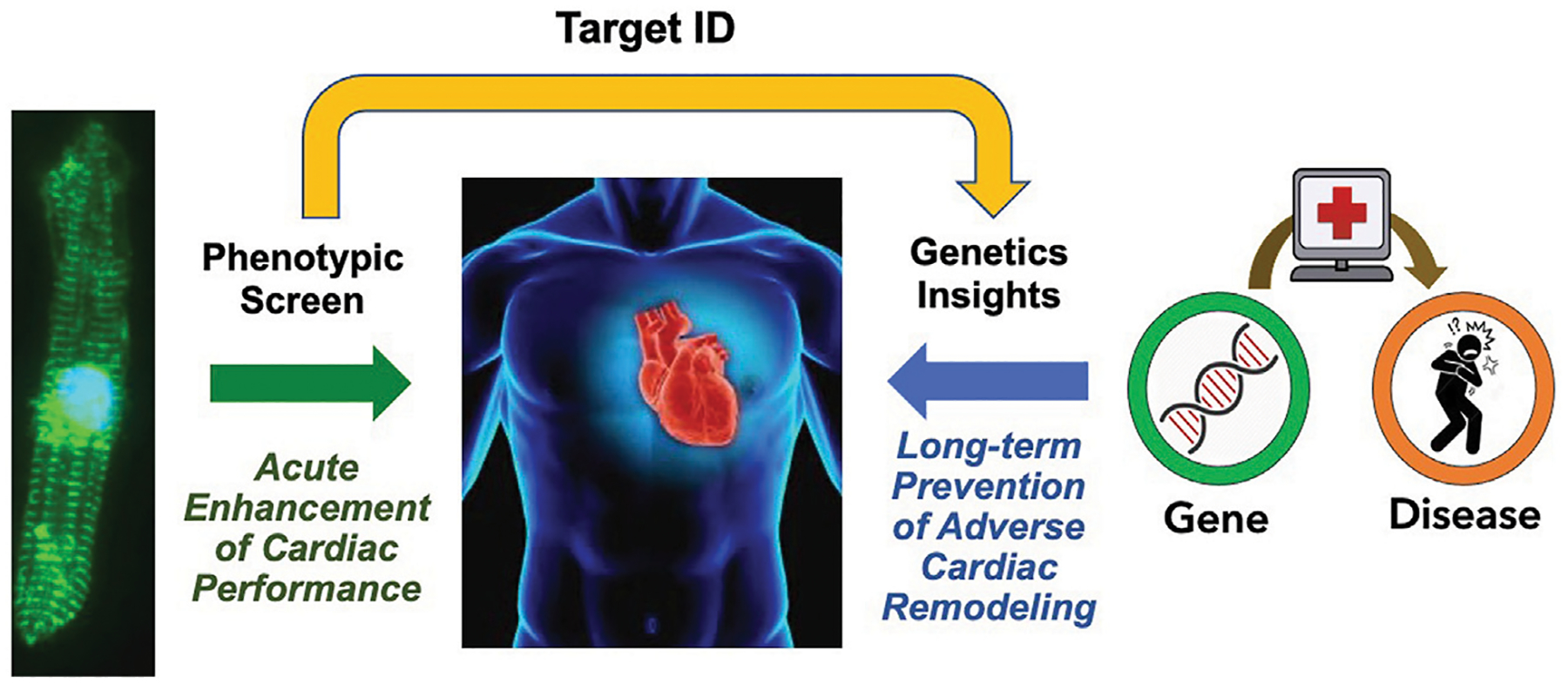
Proposed Path for HF Drug Discovery Bridging the Therapeutic Divide. By combining phenotypic screens to identify agents that improve acute cardiac function, and analysis of association of the target gene associated with the heart failure phenotype to gain insights on the impact of chronic gene perturbation, we may acquire new therapeutics that improve both acute cardiac performance and long-term outcomes.

A unique and fundamental challenge of screening for new HF drugs is that, given the therapeutic paradigm established by the neurohormonal blockade, short-term improvements in cardiac function have largely been uncoupled from long-term outcomes. In contrast to the cancer field, where acute tumor size reduction *in vitro* and *in vivo* presumes long-term clinical benefits, there is no expectation that a drug having acute hemodynamic benefits will have any long-term benefits. One approach to bridge this “translational divide” would be to first carry out an unbiased phenotypic screen for compounds having acute beneficial effects on cardiac function or metabolism, then identify the pharmacological target, and conduct a PheWAS of the target gene to identify any association with HF along with functional genomic studies to surmise the impact of lifelong modulation of the drug target (Figure 2). This approach may help to prioritize candidate molecules with a higher chance of leading to new therapies that acutely improve heart function as well as improve long-term outcomes.

## Expanding the academia-biotech-pharma ecosystem to facilitate cardiovascular drug discovery

Academic centers have long been the home for curiosity-driven biomedical research that is conducted by faculty investigators and trainees. In academia, there is a culture of free-flowing exchange of information and reagents between colleagues across the world. Broad dissemination of knowledge through presentations at scientific meetings and publications is the principal currency of academic researchers. As part of the drug development ecosystem (Figure 1), university-based research plays a critical role in the creation of new knowledge about how genes, cells and molecules function during normal homeostasis and how these mechanisms go awry in human disease. This foundational knowledge, which also includes insights into human genetics and clinical medicine, is the bedrock of target and therapeutic molecule discovery. However, the university setting is traditionally not well-equipped to bridge the gap between their fundamental biological discoveries and the creation of new medicines. On the other hand, the biopharmaceutical industry is adept at the discovery, optimization and clinical development of therapeutic molecules, particularly when the precise molecular target for the drug has already been identified through human genetics and other mechanism-based experimental approaches. While there are teams of highly talented professional scientists in biopharma companies that are passionately engaged in drug discovery, the organizational bandwidth for curiosity-driven research or foundational discovery can often be limited by the incredible demands of maintaining an active pipeline of clinical candidates. Furthermore, scientific insights that are internally generated at biopharma companies are not typically shared publicly at early stages, given the need to protect intellectual property in an incredibly competitive environment. Finally, even the largest pharmaceutical companies can only prosecute a fraction of the early-stage ideas and targets that arise through internal discovery efforts or the published literature, particularly as most of these nascent ideas pose daunting biological uncertainty and technical risks.

While there is a potential for a robust symbiotic relationship between academia and the biopharmaceutical sector, there is a clear need for an intermediate step in translational research that bridges the gap between foundational biological insights and target-based drug development. One important way to address this gap is through the creation of small, venture-funded small biotechnology companies in the cardiovascular field that can de-risk early ideas, create intellectual property, and build toward early proof-of-concept around a therapeutic hypothesis or novel technology. Often, these small biotechs are spun-out from academic laboratories with private investor partnerships, in a model where the inventors and the institution share intellectual property, ownership stake, or future royalties. This model, which encompasses academic laboratories, small biotech spinoffs and larger pharma companies has become commonplace in the oncology therapeutic area, particularly in “hotspot” centers such as Cambridge, MA, the San Francisco Bay Area and San Diego. Other biotechnology hotspots around the world are emerging in Europe and Asia. Despite the fact that cardiovascular disease remains the most common cause of death worldwide, it is noteworthy that the number of small-biotech startups in the cardiovascular area pales in comparison to that in oncology. In the cardiovascular disease area, these small companies will typically require a partnership with a larger company to optimize molecules for clinical testing or perform later-stage clinical trials. Often, large pharmaceutical companies serve as anchor institutions in the local ecosystem and support bio-incubators adjacent to universities and also allocate resources towards their own venture funds.

In order to enhance success in cardiovascular drug development, the entire community of academic, small-biotech and large biopharma scientists must work together to build and nurture an ecosystem that will foster deep mechanistic research, early-stage risk-taking, large pharma “know-how”, and perhaps most importantly, the training of talented and diverse scientists who will be the engines of innovation. Building this culture and infrastructure also has the potential to create job opportunities and new economic growth in regions that are not currently biotechnology hubs. As an important first step in this process, academic laboratories and institutions should foster a culture of entrepreneurship among their faculty and trainees and encourage private sector jobs as important and meaningful career paths that are important for society. Many more cardiovascular investigators should be consulting with local experts in their ecosystem to see if their work can be further de-risked and developed through new company spinouts or partnerships with existing companies. Ultimately, this type of environment will result in a fluid, bi-directional exchange of ideas and talent between academia and the biopharma sector, providing our patients and societies more shots on goal for desperately needed therapies for heart failure and other grievous cardiovascular diseases.

## Concluding remarks

Given the enormous global burden of CVDs, developing new drugs to treat them is a Grand Challenge for our generation. Here, I have highlighted several areas where academia and industry can work together to ensure the successful development of new transformative therapies for CVDs. First and foremost, we need to work toward a better fundamental understanding of human HF, deriving mechanistic insights from rare human diseases and human genetic studies. Examination of residual risk and so-called missing biology will help us identify new therapeutic targets. We also need more predictive preclinical models, and greater emphasis should be placed on the reproducibility of preclinical findings and rigorous vetting of therapeutic hypotheses. The earlier a therapeutic hypothesis is critically evaluated, and the results shared, the better it will be for everyone, reducing collective risks and opportunity costs. New therapeutic modalities, such as modified mRNA and genome editing, offer an unprecedented spectrum of potential targets with extraordinary specificity. Finally, real-world big data projects that use EHRs and genomic data will be valuable for discovering new therapeutic targets, identifying potential regulatory signals, and repurposing existing drugs. Finally, we need to explore new paradigms in which academia and industry can work together more efficiently to deliver needed therapies for the greatest unmet medical need.
